# Genetic variability and discrimination of low doses of *Toxocara* spp. from public areas soil inferred by loop-mediated isothermal amplification assay as a field-friendly molecular tool

**DOI:** 10.14202/vetworld.2016.1471-1477

**Published:** 2016-12-27

**Authors:** Maryam Ozlati, Adel Spotin, Abbas Shahbazi, Mahmoud Mahami-Oskouei, Teimour Hazratian, Mohammad Adibpor, Ehsan Ahmadpour, Afsaneh Dolatkhah, Paria Khoshakhlagh

**Affiliations:** 1 Infectious and Tropical Diseases Research Center, Tabriz University of Medical Sciences, Tabriz, Iran; 2 Department of Parasitology and Mycology, Faculty of Medicine, Tabriz University of Medical Sciences, Tabriz, Iran; 3 Drug Applied Research Center, Tabriz University of Medical Sciences, Tabriz, Iran

**Keywords:** gene flow, Iran, loop-mediated isothermal amplification, polymerase chain reaction, soil, *Toxocara* spp

## Abstract

**Abstract:**

Aim: One of the main diagnostic problems of conventional polymerase chain reaction (PCR) is indiscrimination of low parasitic loads in soil samples. The aim of this study is to determine the genetic diversity and identification of *Toxocara* spp. from public areas soil inferred by loop-mediated isothermal amplification (LAMP) assay.

**Materials and Methods:**

A total of 180 soil samples were collected from various streets and public parks of northwest Iran. The DNA of recovered *Toxocara* eggs were extracted and amplified by PCR and LAMP following ZnSO_4_ flotation technique. The amplicons of internal transcribed spacer-2 gene were sequenced to reveal the heterogeneity traits of *Toxocara* spp. In addition, *Toxocara canis* sequences of southwest Iran were directly retrieved to compare gene flow between two distinct populations.

**Results:**

*Toxocara* spp. eggs were found in 57, 14 and 77 of soil samples using the microscopy, PCR and LAMP (detection limit 1-3 eggs/200 g soil), respectively. 7.7% of isolates were identified as *T. canis* by PCR method, while LAMP was able to detect 27.2%, 15.5% and 12.2% as *Toxocara cati*, *T. canis* and mixed infections, respectively. The kappa coefficient between LAMP and microscopy indicated a strong agreement (0.765) but indicated a faint agreement among LAMP-PCR (0.203) and PCR-microscopy (0.308) methods. A pairwise fixation index (*F*st) as a degree of gene flow was generally low (0.02156) among *Toxocara* populations of northwest and southwest Iran.

**Conclusions:**

The statistically significant *F*st value indicates that the *T. canis* populations are not genetically well differentiated between northwest and southwest Iran. This shows that here is possibly an epidemiological drift due to the transfer of alleles. The LAMP assay because of its shorter reaction time, more sensitivity, and simultaneous detection of environmental contamination to be appears as valuable field diagnosis compared to PCR. Therefore, the detection of low *Toxocara* spp. loads from public area soils will help to expand epidemiological understanding of toxocariasis and establishing preventive strategies in resource-limited endemic of Iran.

## Introduction

Human toxocariasis is a helminthic sapro-zoonotic disease that is caused by the larval stages of common gastrointestinal parasites of dogs (*Toxocara canis*) and cats (*Toxocara cati*) [1,2]. These animals play a notable role in the transmission of toxocariasis, particularly in the tropical, subtropical and temperate regions of the world [3]. Based on host immunity response against parasite and localization of the larvae, four clinical appearances including visceral larva migrans, ocular larva migrans (OLM), neurological toxocariasis, and covert toxocariasis have been defined [3-6]. Human infections, especially children, are frequently acquired using environment contaminated with embryonated *Toxocara* spp. eggs by playing in public areas such as parks, sandpits, and playgrounds [7]. The increasing number of infected stray dogs and cats in public areas, their easy access to unfenced public parks and increasing popularity of keeping animals as pets have caused that rate of sero-prevalence of toxocariasis is unambiguously increasing among healthy children [7]. It is noteworthy that a large number of *Toxocara* eggs in the soil is unusual in sample collected from public places. Even in shelters for dogs and cats, some authors detected up to 10 eggs/50 g sample [8].

Given that the low numbers of eggs in children may cause OLM [9] employing validated diagnostic test should be broadly noticed among neglected hyperendemic foci. One of the principal diagnostic difficulties of single round polymerase chain reaction (PCR) concerning detection of *Toxocara* eggs in soil sources is associated with the presence of inhibitors such as humic acids, lipids, polysaccharides and polyphenols/tannins, which are strongly affected on extracted DNA yield. The accurate identification of *T. canis* and *T. cati* eggs owing to their similar morphology can be difficult to discriminate [10]. A number of PCR-based molecular assays (PCR, PCR-restriction fragment length polymorphism, and real-time PCR) have been conducted for differentiation of *T. canis* and *T. cati* eggs from parks, playgrounds, sandpits, backyards, farmyards gardens, and beaches [11-13]. However, the PCR assays take several hours and necessitate specialized equipment, which make their use impossible in under field conditions. Loop-mediated isothermal amplification (LAMP) has been recently introduced as a rapid and sensitive alternative diagnostic technique in resource-limited endemic regions. The extensive applicability of LAMP has established in the revealing of low parasitic loads in various samples such as *Taenia* spp. eggs in feces samples (detection limit 5 eggs/g of feces) *Schistosoma japonicum* in snails, *Entamoeba histolytica* trophozoites in feces samples (detection limit 1 trophozoite in 500 ml of feces), *Toxoplasma gondii* in water samples, *Leishmania*, *Plasmodium*, and *Dirofilaria immitis* [14-20]. Up to now, only one report is available on the using the LAMP method for detection and discrimination of *T. canis* and *T. cati* eggs from sand samples [21]. The significant prevalence of latent and asymptomatic *Toxocara* infection among young population has identified in northwest Iran; however, there are not many molecular explorations on identification of *Toxocara* spp. in soil samples of Iran [22]. On the one hand, computations of gene flow index among different populations of *Toxocara* spp. could provide a worthwhile data concerning population genetic structures, epidemiological drift of parasite, allele frequencies and speciation.

The aim of this study was to appraise the soil contamination of public areas by *Toxocara* spp. eggs, in the northwest Iran inferred by microscopy, PCR, LAMP and phylogenetic strategies to identify the accurate prevalence rate, epidemiological drift and genetic variability and precise taxonomic status of parasite in the region.

## Materials and Methods

### Ethical approval

This research was approved by the Faculty of Medicine, Tabriz University of Medical Sciences, Tabriz, Iran.

### Survey area, collection and recovery of soil sample

This examination was conducted from February 2014 to May 2015 in Tabriz city located at Northwest Iran. Tabriz as the second largest city in Iran with 1,800,000 populations has a cold to semi-arid climate with regular seasons. The average annual rainfall and temperature are 280 mm and 12.6°C, respectively. A total of 180 soil samples were collected from five divisions (north, south, east, west and center) of the city covering public parks and streets. Each sample (approximately 200 g soil) was collected from 3 cm depth of soil and placed in labeled polyethylene bags.

### Microscopic observation and floatation technique

After 1-2 days, the dried soil samples were sieved through a 0.5 mm mesh. To recovery of *Toxocara* eggs from contaminated soils, the zinc sulfate (ZnSO_4_) flotation was employed according to modified Dada method [23]. In brief, 10 g samples with 50 ml of 0.1% Tween 80 were vortexed for 30 min. The supernatant free of eggs was decanted and a saturated flotation solution (ZnSO_4_, 1.52 specific gravity) was added to the tubes containing the remaining sediment and centrifuged at 1500 rpm for 10 min. The solution was added to form a meniscus and a cover slip was overlaid. After 15 min, the cover slip was transferred to a glass slide and was examined at a magnification of 40× and 100× for *Toxocara* eggs based on morphological characters such as presence of larva inside the pitted eggs. Furthermore, unembryonated eggs were incubated in 0.5% formalin at 25°C for more than 2 months.

### DNA extraction and PCR amplification

The total DNA from all flotation fluid was extracted using the Takapozist DNA extraction kit according to the manufacturer’s instructions. First, eggs were sonicated for 5 times in 20 s. The homogenized samples were subjected through the freeze-thawing technique (10 cycles of freezing [10 min at liquid nitrogen] and thawing [10 min at 95°C]) and proteinase K digestion was performed overnight. To identify *Toxocara* spp. the species-specific primers were chosen from internal transcribed spacer 2 (ITS2) gene that were previously described as Tcan1 (5’-AGTATGATGGGCGCGCCAAT-3’) and NC2 (5’-TAGTTTCTTTTCCTCCGCT-3’) for *T. canis*, and Tcat1 (5’-GGAGAAGTAAACTC-3’) and NC2 for *T. cati* [12]. The PCR amplification was carried out in 25 µl reaction volumes containing 0.3 µl (5 u/µl) of Taq DNA polymerase (Cinnagen, Iran), 2.5 µl of ×10 PCR buffer (Cinnagen, Iran), 0.9 µl (50 mM) MgCl_2_ (Cinnagen, Iran), 0.5 µl (10 mM) of deoxynucleotide triphosphate (dNTP) Mix (Cinnagen, Iran), 10-13 µl deionized distilled water, 1 µl of each forward and reverse primers (15 pmol), 3-4 µl of bovine serum albumin 0.1% as enhancer, and 3-4 µl of DNA template. Amplifications were performed under the following conditions: Initial cycle at 94°C for 30 s, followed by 30 cycles of denaturation at 94°C for 60 s, annealing at 55°C for 30 s and extension by polymerase at 72°C for 30 s and a final cycle at 72°C for 5 min. The PCR products were electrophoresized on 1% (W/V) agarose gel stained with DNA safe stain.

### LAMP assay

The forward and backward external primers (F3 and B3) and forward and backward internal primers (FIP and BIP) of ITS2 gene were used to perform LAMP assay based on Macuhova *et al*. study [21]. The LAMP assay was conducted in 25 µl of a reaction mixture consisting of 40 pmol/uL concentration of each inner primer (FIP and BIP), 5 pmol/uL concentration of each outer primer (F3 and B3), 8 U *Bst* 2.0 DNA polymerase (New England Biolabs), 1 µl SYBR Green I, 2.5 µl ×10 buffer, 1.4 mM of dNTPs, 3 mM of MgSO_4_, 0.8 M of betaine, and 1 µl of template DNA. The mixture was incubated at 64°C for 60 min in a heating block and then heated at 80°C to terminate the reaction. A positive control of *Toxocara* DNA and water as a negative sample were included in each LAMP assay. At the end of incubation, the presence of the target gene was characterized by the presence of white turbidity of magnesium pyrophosphate which detected visually by the naked eye. Accuracy of the findings was confirmed by both electrophoresis and fluorescence detection. LAMP products were electrophoresized on 1.5% agarose gel and were observed under ultraviolet (UV) light after staining by safe stain for 30 min. Positive samples showed the typical ladder pattern which was not a single band. For fluorescence detection, 1 µl of SYBR Green I was added on LAMP products and were irradiated with a UV lamp and photographed. The presence of fluorescence indicated the presence of the target gene. To compare the analytical sensitivity between LAMP and PCR assays, the various numbers of *Toxocara* eggs (1-6 eggs/200 g soil) were evaluated.

### DNA sequencing, alignment and phylogenetic analysis

To confirm the specificity of the LAMP primers, a single round PCR was done based on B3 and F3 outer primers. Amplicons of the ITS2 gene were purified with the Wizard SV Cleanup System (Promega). ABIPRISMTM 3130 Genetic Analyzer automated sequencer (Applied Biosystem, USA) directly sequenced PCR products from 10 randomly selected samples. Contigs (overlapped sequences) from all samples were aligned and edited at consensus positions compared to GenBank sequences of all regional species using Sequencher Tmv.4.1.4 Software for PC (Gene Codes Corporation). *T. canis* sequences of southwest Iran (Accession nos.; AB743614-AB743617 and AB819327-AB819330) were directly retrieved from GenBank database (FASTA format). The sequences pairwise distances (percent identity and divergence) between sequenced isolates and other country sequences were constructed using the MegAlign program from Laser Gene Bio computing Software Package (DNASTAR, Madison, WI). MEGA 5.05 software with maximum likelihood algorithm and Kimura2-parameter model were used in order to construct phylogeny tree. The diversity (haplotype and nucleotide diversity), neutrality indices (Tajima’s D and Fu’s Fs statistic) and fixation index (*F*st) were estimated by DnaSP software version 5.10 [24].

### Statistical analysis

Statistical analysis was performed using SPSS version 16.0 software. The Student’s t-test was used to compare the frequencies of *Toxocara* prevalence among city areas at a confidence interval (CI) of 95%. The degree of agreement among diagnostic methods results was determined by Kappa (κ) value with 95% CIs.

## Results

**Figure-1:**
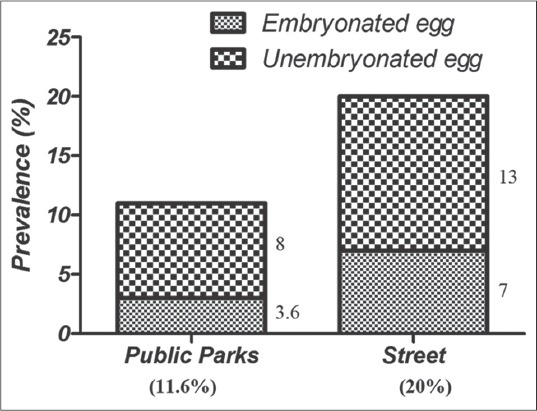
Prevalence of embryonated/unembryonated *Toxocara* eggs in the soil samples of streets and public parks recovered by the parasitological method.

**Figure-2:**
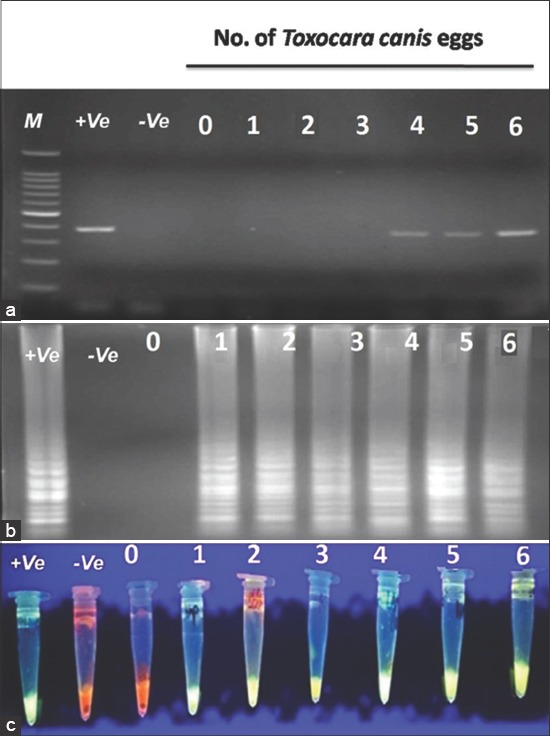
Sensitivity evaluation of loop-mediated isothermal amplification (LAMP) and polymerase chain reaction (PCR) assays based on number of *Toxocara canis* eggs in soil samples. (a) DNA amplification of *Toxocara canis* eggs by PCR on a 1.5% agarose gel (detection limit >3 eggs/200 g soil), (b) agarose gel electrophoresis of LAMP products (detection limit 1-3 eggs per 200 g soil), (c) LAMP, visual detection by fluorescence. M=100 bp DNA ladder marker; +Ve: Positive control; −Ve: Negative control (water).

**Figure-3:**
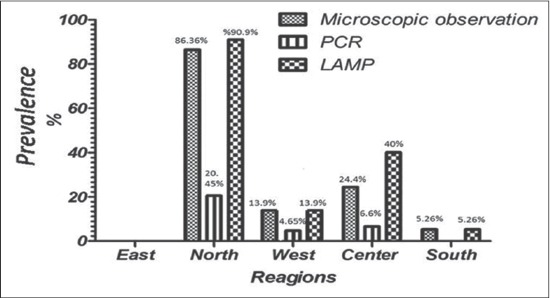
Prevalence of *Toxocara* spp. infection inferred by microscopic, polymerase chain reaction and loop-mediated isothermal amplification assays in various geographical regions of Tabriz.

**Figure-4:**
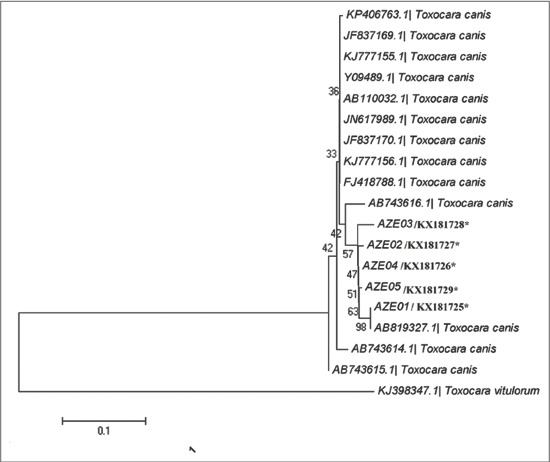
The phylogeny tree of *Toxocara canis* isolates according to the maximum-likelihood was conducted based on the multiple sequence alignment of internal transcribed spacer 2 gene by MEGA5.05. Distance represents the number of base substitutions per site. *Toxocara vitulorum* is the out-group branch.

**Figure-5:**
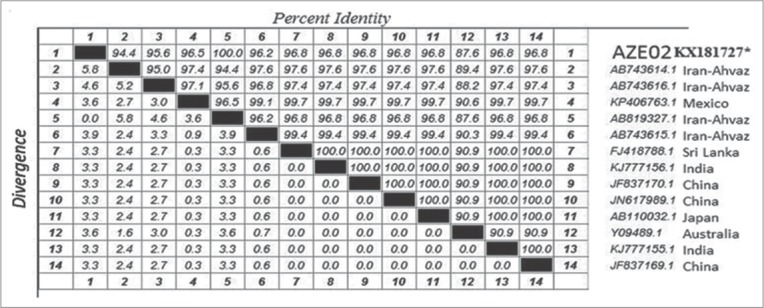
The percent of diversion and identity between the identified *Toxocara canis* (AZE02; KX181727*) and selected references’ sequences circulating globally from GenBank database inferred by partial internal transcribed spacer 2 gene.

The findings of microscopy, PCR and LAMP methods are presented in Table-1. 57 (n=31.6%) of 180 soil samples identified by microscopy which the highest and lowest prevalence rate were belonged to north (21.1%) and east (0%) regions, respectively. As well, 7.7% (n=14) samples were dominantly diagnosed to *T. canis* by PCR method while no *T. cati* infection was found by PCR. The kappa coefficient between PCR and microscopy indicated a faint agreement (0.308) and this difference was not found to be statistically significant (Pv=0.745). 77 (42.7%) of 180 soil samples were contaminated to *T. cati* (n=49: 27.2%), *T. canis* (n=28: 15.5%) (Table-1) and mixed infections (n=22: 12.2%, not shown in Table-1). The kappa coefficient between LAMP and microscopy indicated a strong agreement (0.765, Pv=0.01) but indicated a faint agreement among LAMP-PCR (0.203, Pv>0.05). The frequency of embryonated and unembryonated eggs recovered from streets and public parks is shown in Figure-1. The highest rate of fully embryonated eggs observed in the streets (13%). The majority number of identified *T. cati* and *T. canis* eggs were ranged 1-3 and 1-10/200 g soil, respectively. The analytical sensitivity between LAMP and PCR assays were assessed based on number of *Toxocara* eggs/200 g soil (Figure-2). Findings show that PCR was able to distinguish more than 3 eggs/200 g soil (Figure-2a) while the LAMP detected 1-3 eggs/200 g soil sample (Figures-2b and 2c). Using species-specific primers, the PCR method showed 380 bp fragment from *T. canis* (Figure-2a), however the 370 bp fragment did not amplify for *T. cati* isolates. The frequency of *Toxocara* spp. identified by employed diagnostic methods is demonstrated in various divisions of Tabriz city according to Figure-3. Phylogenetic analysis revealed that the *T. canis* (AZE01-AZE05; deposited at the GenBank under accession numbers; KX181725-KX181729) grouped in its specific complex and *Toxocara vitulorum* (Accession number: KJ398347) was considered as an out-group branch (Figure-4). The percent identity (ranges: 96.5-100%) and divergence (ranges: 0-5.6%) among identified isolate (AZE02) and other countries’ sequences shown in Figure-5. Multiple sequence alignment of 10 *T. canis* isolates revealed two new haplotypes (AZE02 and AZE03) with low diversity values (haplotypes diversity; 0.295, nucleotide diversity; 0.00088) and high neutrality values (Tajima’s D; 0.71434 and Fu’s Fs; 0.659) (Table-2). *F*st as a degree of gene flow was generally low (0.02156) between *Toxocara* populations of northwest and southwest Iran.

**Table-1:** Comparison of parasitological and molecular methods for the detection of *Toxocara* sp. eggs in different regions of Tabriz, northwest Iran.

Region	Studied districts	Number of examined soil samples	Floatation and microscopic observation (%)	Molecular analyses (%)			

				PCR by ITS2-gene		LAMP by ITS2-gene	
	
				*T. cati*	*T. canis*	*T. cati*	*T. canis*
Northwest Iran/Tabriz	East	10	0 (0)	0	0	0	0
	South	38	2 (1.11)	0	0	0	2 (1.11)
	West	43	6 (3.33)	0	2 (1.11)	4 (2.22)	2 (1.11)
	North	44	38 (21.1)	0	9 (5)	38 (21.1)	13 (7.2)
	Center	45	11 (6.1)	0	3 (1.66)	7 (3.8)	11 (6.1)
	Total	180	57 (31.6%)	0	14 (7.7%)	49 (27.2%)	28 (15.5%)
						77 (42.7%)	

ITS=Internal transcribed spacer 2, PCR=Polymerase chain reaction, LAMP=Loop-mediated isothermal amplification

**Table-2:** Diversity and neutrality indices of *T. canis* based on nucleotide sequences of ITS2-rDNA gene in northwest and southwest Iran.

Region	Diversity indices				Neutrality indices	
	
	n	Hn	Hd±SD	Nd (p)±SD	Tajima’s D	Fu’s Fs statistic
Northwest Iran (Tabriz)	10	2	0.295±0.155	0.00088±0.00077	0.71434	0.659
Southwest Iran (Ahvaz)	8	2	0.345±0.172	0.00093±0.00088	0.69432	0.654

ITS2=Internal transcribed spacer 2, Hn=Number of haplotypes, Hd=Haplotype diversity, Nd=Nucleotide diversity, *T. canis*=*Toxocara canis*, SD=Standard deviation

## Discussion

In current inquiry, the high prevalence rate (42.7%) of low *Toxocara* spp. loads was detected and identified by LAMP assay in soil samples of northwest Iran where despite the meaningful increasing sero-prevalence of *Toxocara* infection (3% to 29.04%) among young population [25-28] here is no inclusive examination on phylo-molecular epidemiology of infected soil samples yet. Up to now, several epidemiological surveys have been focused on contamination rate of public areas to *Toxocara* egg [29,30]. The global prevalence of *Toxocara* spp. in public parks estimated to be 9.75%, 6.73%, 28.31%, 11.57%, 0.55%, 14.03%, and 11.87% in North America, Latin America, Europe, Asia, Middle East, Australia, and Turkey, respectively [29,30]. Using zinc sulfate flotation technique, 31.6% of soil samples were contaminated with *Toxocara* eggs, which was higher than some formerly investigations accomplished in different sites of Iran. Epidemiological studies conducted in Iran demonstrate the contamination rate of public parks has reported to be 6.3%, 3.9%, 30.4% and 10% in Shiraz (south), Urmia (northwest), Ahvaz (southwest) and Tehran (central) cities, respectively [22,31-33]. These differences may justify by several facts including socioeconomic status, geographical parameters, poor sanitation, sample size, climatological variables, the number of stray cats and dogs, and especially employing various diagnostic tools. Furthermore, in this study, the majority of soil samples were collected from unfenced parks, which this could be provided the easy access of stray infected hosts.

A similar study conducted in Turkey has exhibited the high prevalence of soil contamination in unfenced public parks than fenced parks [30]. Hence, using antiparasitic drugs, enforcement of hygiene programs in park, management of stray dogs and cats, and increasing public awareness by municipal government can be decreased the prevalence of *Toxocara* infection.

In this study, the ratio of *T. canis* to *T. cati* was approximately found to be 1:2 (0.57) by the LAMP method, which is in conflict with other reports [22]. This shows that stray cats and dogs are the main contaminating animals in the region. However, our ongoing project on pet contamination showed that 11% of these animals are infected to *T. canis* (data not published yet). In this study, only 7.7% (n=14) of soil samples were diagnosed by PCR as *T. canis* (detection limit more than 3 eggs/200 g) while 54.9% of samples were diagnosed as *T. cati* (27.2%) and *T. canis* (15.5%) and mixed infections (12.2%) by LAMP (detection limit 1-3 eggs/200 g). This shows that PCR is less sensitive than the LAMP assay. Theses discrepancies are described by some following facts: First, the activity of Taq DNA polymerase is inhibited by soil components such as humic acids, lipids, polysaccharides, and polyphenols/tannins while the *Bst* 2.0 DNA polymerase to overcome potential inhibitors in extracted DNA templates. Second, it is suggested that in a low *Toxocara* egg burden (particularly in *T. cati* egg), a considerable amount of parasite DNA is lost during the extraction and purification processes which in this case, a set of PCR primers cannot be specifically annealed and amplified target templates. They can interfere with the reaction at several levels, leading to different degrees of attenuation and even to complete inhibition [34].

Based on sequencing and phylogenetic findings, only two new haplotypes (AZE02 and AZE03, haplotype diversity: 0.295, homology: 96.5-100%) was identified among *T. canis* isolates. Low genetic diversity (HD; 0.295) of *T. canis* isolates can explain to be conserved the nature of ITS2 gene and its high copy number or small effective sample size [35]. In addition, this may be related to fertilization of *Toxocara* adult worm and/or to the longevity of the parasite in the stray dogs [36].

In this study, the *F*st index was generally low (0.02156) among *T. canis* populations of northwest and southwest Iran. The statistically significant *F*st value indicates that the *T. canis* populations are not genetically well differentiated among mentioned regions. This shows that here is possibly an epidemiological drift due to transfer of alleles from one population to another population or vice versa.

## Conclusion

The LAMP method because of its shorter reaction time, more sensitivity, simultaneous detection of environmental contamination and visual discriminatory of positivity to be appears as valuable alternative tool compared to PCR. Therefore, the detection of low *Toxocara* spp. loads from public district soils will help to expand epidemiological understanding of toxocariasis and employing preventive strategies in resource-limited endemic of Iran. Furthermore, *F*st value in microevolutionary scale reflects new insights for further exploration in characterizing the local transmission patterns of *Toxocara* populations among various foci of Iran.

## Authors’ Contributions

AS and MO and TH: Contributed to the acquisition of data carried out the molecular genetic studies and have been involved in drafting the manuscript. ASh and AD and MMO: Participated in the design of the study, contributed to sample collection and helped to draft the manuscript. MA and PK and EA: Performed the statistical analysis and have been involved in critically revising the manuscript for important intellectual content. All authors read and approved the final version of the manuscript.
